# Extranasopharyngeal angiofibroma: clinical and radiological presentation

**DOI:** 10.1007/s00405-012-2041-4

**Published:** 2012-05-15

**Authors:** Anna Szymańska, Marcin Szymański, Kamal Morshed, Elżbieta Czekajska-Chehab, Małgorzata Szczerbo-Trojanowska

**Affiliations:** 1 Department of Interventional Radiology and Neuroradiology, Medical University of Lublin, Ul. Jaczewskiego 8, 20-954 Lublin, Poland; 2 Department of Otolaryngology, Medical University of Lublin, Ul. Jaczewskiego 8, 20-954 Lublin, Poland; 3 Department of Radiology, Medical University of Lublin, Ul. Jaczewskiego 8, 20-954 Lublin, Poland

**Keywords:** Extranasopharyngeal angiofibroma, CT, MRI, Symptoms

## Abstract

Nasopharyngeal angiofibroma (NA) is a rare, vascular tumor affecting adolescent males. Due to aggressive local growth, skull base location and risk of profound hemorrhage, NA is a challenge for surgeons. Angiofibromas have been sporadically described in extanasopharyngeal locations. We review ten cases of extranasopharyngeal angiofibroma (ENA) and discuss the incidence, clinical presentation and management of this pathology. The group consisted of 4 males and 5 females aged 8–49. There were 7 patients with nasal angiofibroma, 1 patient with laryngeal angiofibroma, 1 patient with oral angiofibroma and another patient with infratemporal fossa tumor. In patients with nasal angiofibroma most common presenting symptoms were nasal obstruction and epistaxis. Patients with laryngeal angiofibroma suffered from mild dysphagia and patients with the infratemporal fossa tumor had painless cheek swelling. In four patients with nasal tumor computed tomography (CT) demonstrated mass with strong to intermediate contrast enhancement. In one patient with nasal tumor carotid angiography demonstrated pathological vessels without intensive tumor blush. Infratemporal fossa tumor showed intensive contrast enhancement on CT and magnetic resonance imaging (MRI) scans, and abundant vascularity on angiography. Laryngeal and oral angiofibroma required no radiological imaging. Three nasal tumors were evaluated before introduction of CT to clinical practice. All patients underwent surgery. No recurrences developed. ENAs differ significantly from NAs regarding clinical and radiological presentations. They lack typical clinical and radiological features as they develop in all age groups and in females, may be less vascularised, arise from various sites and produce a variety of symptoms.

## Background

Nasopharyngeal angiofibroma (NA) is a rare vascular tumor, which represents 0.05 % of all head and neck tumors. At the same time, it is the most common benign neoplasm of the nasopharynx [1]. NA occurs predominantly in adolescent males. Although histologically benign it shows locally aggressive growth with bone destruction and spread through natural foramina and fissures. It originates from the posterolateral wall of the nasopharynx and from this site usually extends to the nasopharynx, nasal cavity, paranasal sinuses, spheno-palatine foramen and infratemporal fossa. In 10–20 % of the cases tumor invades the cranial cavity [2].

Extranasopharyngeal angiofibromas (ENAs) have been sporadically described in the literature. In this report, we present ten cases of extranasopharyngeal angiofibroma arising from various sites and discuss the incidence, clinical presentation and management of this pathology in comparison to NAs.

## Materials and methods

From March 1967 to June 2007, ten patients with histologically proven extranasophryngeal angiofibroma were treated at our institution. There were 6 males and 4 females aged 8–49. Age range and median in males (12–49 years, median 27) and females (8–47, median 29) were comparable. All patients had primary tumors. In all patients tumor was removed surgically. Histopathological examination of postoperative samples was reexamined and confirmed the diagnosis of angiofibroma. A retrospective chart analysis of ENA patients was performed. We analyzed the point of origin, clinical presentation, radiological features and treatment of tumors in the presented group. We also compared our findings with typical features of angiofibromas of nasopharyngeal origin.

## Results

There were seven patients with nasal angiofibroma. In all cases, tumor aroused from the nasal septum. One patient had a laryngeal angiofibroma originating from the epiglottis and another patient had a tumor originating from the palatine tonsil. In another patient, tumor arising from the fossa pterygo-palatine spread to the infratemporal fossa and cheek with no invasion of the nasopharynx.

Clinical examination revealed a firm, painless mass in all patients with nasal tumor. The patient with the laryngeal angiofibroma was a 8-year-old girl with a 4-week history of mild dysphagia. Examination revealed a smooth, round, reddish lesion, which was located on the top of the epiglottis. The tumor was covered with mucosa and showed some mobility during breathing and phonation. No other pathological findings were detected. In the patient with oral angiofibroma the tumor was a smooth, painless lump, covered with normal mucosa, located on the right palatine tonsil. The patient with angiofibroma of the infratemporal fossa presented with painless swelling of the cheek. In the whole group, the delay between the onset of symptoms and the surgery ranged from 1 month to 1 year.

**Fig. 1:**
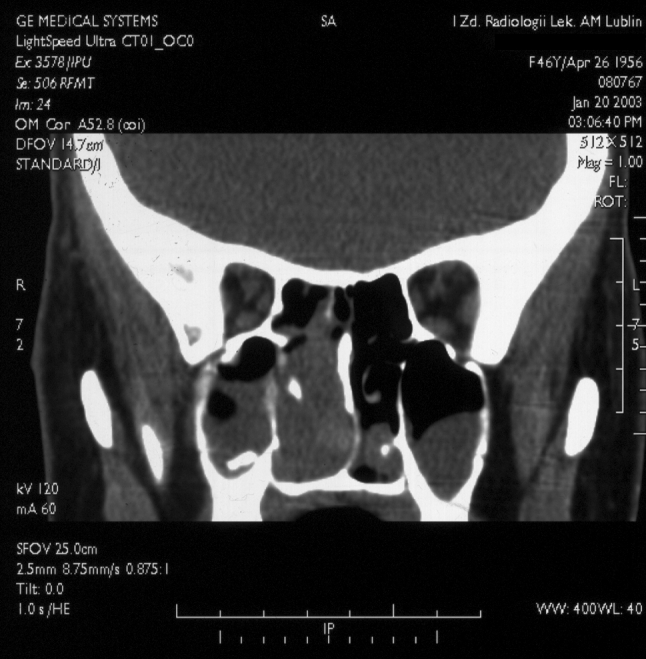
Computed tomography, coronal plane, shows homogenous tumor mass in the right nasal cavity

**Fig. 2:**
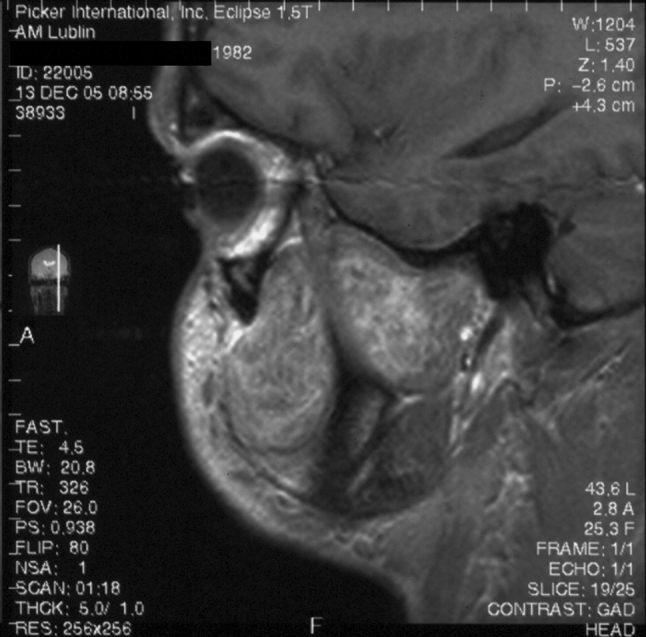
Magnetic resonance, saggital T1-weighted image after contrast administration. Large tumor in the infratemporal fossa and cheek shows intensive, inhomogenous contrast enhancement [25]

Three patients with nasal angiofibroma were diagnosed on the basis of the presented symptoms, clinical examination and plain radiograms, before the introduction of computed tomography (CT) into clinical practice at our institution. Four patients with nasal tumor underwent CT, which demonstrated homogenous mass, with contrast enhancement ranging from strong to intermediate (Fig. 1). In one case, signs of bony destruction with tumor invasion to the ethmoid sinus were visible. One patient with nasal tumor underwent carotid angiography, which demonstrated pathological vessels, without intensive, hypervascular blush typical for NA. The patient with the tumor of the infratemporal fossa underwent CT, magnetic resonance imaging (MRI) and carotid arteriography with preoperative embolization. The lesion showed intensive contrast enhancement on CT and MRI as well as signal-void areas on MR images, typical for high flow vessels (Fig. 2). Arteriography revealed abundant vascularity with main blood supply from the internal maxillary artery. In the cases of laryngeal angiofibroma and angiofibroma of the tonsil tumors were small and well visible on clinical examination, and did not require radiological imaging. In thee patients, additional biopsy of the lesion was performed elsewhere and was followed by no significant bleeding.

All patients underwent surgery. Tumors originating from the nasal cavity were removed endonasally. The tumor arising from the palatine tonsil was removed endorally. For epiglottic angiofibroma transoral laser CO_2_ surgery was applied. Cervical approach with temporary mandible split was used to remove extensive infratemporal fossa tumor.

Apart from the patient with the infratemporal fossa tumor, there was no significant intraoperative bleeding. The postoperative course was uneventful. No recurrences developed.

Detailed information of the described cases is presented in the Table 1.

**Table 1:** Detailed information of the presented cases

No./ID/sex/age	Site/point of origin	Symptoms	Duration	Radiological findings	Treatment/approach
1./JK/M/31	Left nasal cavity/nasal septum	Nasal obstruction, rhinolalia	3 months	–	Surgery, 1967
2./MM/M/42	Right nasal cavity/nasal septum	Epistaxis, nasal obstruction, rhinolalia	4 months	–	Surgery, 1969
3./EB/F/18	Left nasal cavity/nasal septum	Nasal obstruction, epistaxis, rhinolalia,	2 months	–	Surgery, 1970
4./TC/F/40	Right nasal cavity, ethmoid sinus/nasal septum	Nasal obstruction, epistaxis, headache, mucopurulent discharge	12 months	CT: tumor in the nasal fossa with bony erosion and invasion of the ethmoid sinus	Surgery, 1976
5./GJ/M/12	Right nasal cavity/inferior turbinate	Nasal obstruction, epistaxis, mucopurulent discharge	1 month	CT: tumor in the nasal vault DSA	Surgery, 1987
6./RB/M/15	Right nasal cavity/nasal septum	Nasal obstruction, epistaxis	3 months	CT: tumor in the nasal fossa	Surgery, 1998
7./UB/F/47	Right nasal cavity, ethmoid sinus/nasal septum	Nasal obstruction	4 months	CT: tumor in the nasal fossa	Surgery, 2003
8./PK/F/8 [27]	Larynx/epiglottis	Dysphagia	1 month	–	Surgery, 2004
9./MW/M/23 [25]	Left infratemporal fossa, cheek/fossa pterygo-palatine	Swelling of the cheek	12 months	CT/MR: tumor in the pterygo-palatine and infratemporal fossa, cheek. Arteriography: intensive blush	Surgery, 2006
10./JK/M/49	Right palatine tonsil	Dysphagia	1 month	–	Surgery, 2008

*DSA* Digital subtraction angiography

**Fig. 3:**
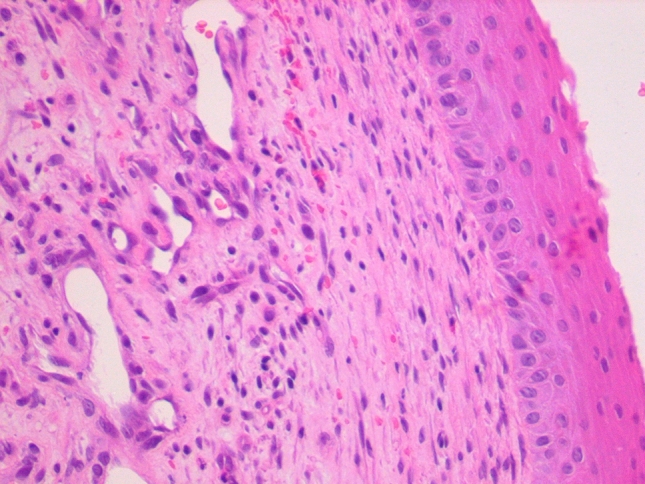
Histologic section of the infratemporal fossa tumor (H&E stain, magn. ×400) shows fibrous stroma with ectatic, thin-walled vascular channels [25]

In four cases (numbers 3, 5, 6 and 9 in Table 1), histopathological appearance was typical for NA with predominance of the vascular component in the fibrous stroma (Fig. 3). Three of them are young boys which may suggest similar etiology to juvenile angiofibroma. Histopathology in the rest of the described patients was more heterogeneous with different relations between fibrous and vascular component.

## Discussion

Angiofibromas of the head and neck in most cases originate from the nasopharynx. The etiology of the nasopharyngeal angiofibroma remains unclear. Strong sex and age correlation suggests the contribution of hormonal disorders of the pituitary androgen–estrogen system. However, there is still no direct evidence proving this hypothesis. The tumor affects almost exclusively males and the symptoms occur at the mean age of 13–15 years [1, 3]. The incidence of the NA in females and other age groups has been reported, but still raises controversy [4–6]. NA shows a number of characteristic features in regard to clinical manifestation and radiological image. Typical patient is a young boy with progressive nasal obstruction and recurrent epistaxis [7]. Classic radiological picture of this tumor is based on its predictable growth pattern and abundant vascular component. NA arises from the superior margin of the spheno-palatine foramen and grows into the nasopharynx and laterally into the pterygo- palatine fossa. The typical radiological findings include strongly enhancing nasopharyngeal mass, widening of the pterygo-palatine fossa, erosion of the pterygoid process of the sphenoid bone [8, 9]. Extension into the nasal fossa, infratemporal fossa, invasion of the paranasal sinuses with destruction of their walls may be present at later stages of the disease.

Extranasopharyngeal angiofibroma is a rare finding. In a recent review of the literature (1918–2002), Windfuhr and Rembert [10] found 58 reports of this entity describing 65 cases.

There were eight more cases reported in the literature since then [11–18].

These tumors present different clinical features from the NAs. They occur more frequently in females, which accounts for 25–26 % of all cases [10, 14, 19]. They also develop in older age group than NA, with the mean age of presentation 22 years [10, 16, 19, 20]. In our study, the mean age of presentation was 28 years. However, cases of angiofibromas at atypical sites in young children have occasionally been described. Schick et al [21] reported a case of 15-month-old boy with tumor located close to the lacrimal sac. In our study, females constituted a half of the group. The youngest patient was 8-year old, which is uncommon, as only 8 of 73 extranasopharyngeal angiofibromas described in the literature were observed in the first decade of life [10–18].

Extranasopharyngeal angiofibromas have been reported to arise from various sites in the head and neck region. The most common point of origin is the maxillary sinus (24.6 %) [10, 14]. The majority of patients in our study had a tumor in the nasal cavity originating from the nasal septum, with invasion of the ethmoid sinus in two cases [10, 18]. There are also reports of tumors located in the ethmoid sinus, nasal cavity, nasal septum, larynx, sphenoid sinus, cheek, conjunctiva, oropharynx, retromolar area, middle turbinate and inferior turbinate [10–14, 16, 20, 22–24]. One patient in our study had a tumor originating from the tonsil, which is uncommon, as there were only two such cases reported in the literature. Cases of tumors in the following locations have been also described: external nose, hard palate, external ear, lacrimal sac, carotid bifurcation, esophagus, trachea, facial nerve, middle cranial fossa and infratemporal fossa [10, 21, 25, 26]. In our study there was one patient with extensive tumor of the infratemporal fossa and cheek. In four cases of laryngeal angiofibromas reported in the literature, tumors were located in the aryepiglottic fold, vocal cord, interarytaenoid region and in the laryngo-pharyngeal junction. To our knowledge, our 8-year-old patient with angiofibroma originating from the epiglottis is the first described case of angiofibroma arising in such location [27].

Unlike NAs, symptoms of extranasopharyngeal angiofibromas are non-specific and numerous. Clinical manifestation and the onset of symptoms depend on the tumor site. Tumors originating from the nasal cavity may be diagnosed relatively early due to limited space for tumor growth and symptoms characteristic for NA. In our observation, nasal obstruction and epistaxis occurred, respectively, in six and five out of seven patients with nasal angiofibroma. Less specific symptoms like rhinolalia, headache and mucopurulent nasal discharge were stated in six cases*.* Clinical presentation of laryngeal lesions includes hoarsness, dysphagia, dyspnea and stridor [23]. In our patient with tumor arising from the epiglottis, the only symptom was minor dysphagia, probably due to the early stage of the disease. ENAs located in other regions may have more confusing clinical presentation and the delay between the onset of symptoms and the diagnosis may be longer, as it has been the case in the patient with the infratemporal fossa tumor. Tumors growing in paranasal sinuses may manifest with pain, fever, rhinorrhea, swelling of the cheek, proptosis, headache, progressive nasal obstruction, occasional epistaxis [10, 22].

Histopathological appearance typical for NA consists of numerous wide, irregular vessels with a single layer of endothelial cells, embedded in fibrous stroma. The abundant vascular component is responsible for excessive bleeding during surgery or following biopsies. It also contributes to certain characteristic radiological features of NAs, including strong contrast enhancement on CT and MR images, signal-void areas representing tumor vessels visible on MR images, as well as intensive vascular blush demonstrated on angiography [8, 28]. CT and/or MRI and carotid angiography are required for optimal preoperative evaluation of NAs. Selective angiography is a useful diagnostic method to demonstrate tumor vascular composition and confirms the diagnosis. It also allows tumor embolization, which reduces intraoperative bleeding. Due to a risk of profound hemorrhage, in a presence of characteristic clinical symptoms and classic radiological findings, preoperative biopsy is not recommended in the management of NAs.

According to histopathology ENAs constitute a more heterogeneous group. The predominance of the vascular component in the fibrous stroma characteristic for NA has been stated only in four patients with ENA in the analyzed group. Therefore, classic radiological findings characterizing NAs are not shared by ENAs. Most ENAs enhance after contrast medium injection, however, enhancement is not a constant sign, and in our study varied from intermediate to strong [10, 22–24]. Unlike NAs, radiological presentation of extranasopharyngeal angiofibromas is much more variable also due to their various locations. From their point of origin, tumors may spread to adjacent areas by widening of natural foramina and fissures or by erosion of bony structures, which is well demonstrated on CT [19, 22, 26]. In our study, CT performed in four patients with nasal angiofibroma in one case revealed tumor extension to the ethmoid sinus.

Extensive angiofibroma of the infratemporal fossa presented on CT and MRI radiological features characteristic for highly vascularized lesion. Carotid angiography showed multiple tumor vessels with main blood supply from the internal maxillary artery. However, lack of hypervascularity on angiograms does not exclude the diagnosis of the ENA [10], which has been the case in one of our patients with nasal tumor. The blood supply to the ENA depends on its point of origin and location, whereas in NAs the main feeder is the maxillary artery.

As for NAs, the treatment of choice for ENAs is surgery. Radiotherapy may be applied for unresectable lesions. Surgical approach is tailored to the location and size of tumor. Despite benign histopathological nature, NAs present a challenge to a surgeon due to aggressive growth, intensive intraoperative bleeding and a recurrence rate ranging from 6 to 27.5 % [29]. Apart from the tumor of the infratemporal fossa, all tumors in our study were relatively small and their resection was not accompanied by significant bleeding. There were no recurrences in the evaluated group. No recurrences of ENAs were reported in the literature [10].

In conclusion, ENAs differ significantly from NAs regarding clinical and radiological presentations. They lack typical clinical and radiological features as they develop in all age groups and in females, arise from various sites, may be less vascularised and produce a variety of symptoms depending on the point of origin.
